# Extremely rapid isotropic irradiation of nanoparticles with ions generated in situ by a nuclear reaction

**DOI:** 10.1038/s41467-018-06789-8

**Published:** 2018-10-26

**Authors:** Jan Havlik, Vladimira Petrakova, Jan Kucka, Helena Raabova, Dalibor Panek, Vaclav Stepan, Zuzana Zlamalova Cilova, Philipp Reineck, Jan Stursa, Jan Kucera, Martin Hruby, Petr Cigler

**Affiliations:** 1 Institute of Organic Chemistry and Biochemistry of the CAS, Flemingovo nam. 2, 166 10 Prague 6 Prague, Czech Republic; 2 Faculty of Science, Charles University, Hlavova 2030, 128 40 Prague 2 Prague, Czech Republic; 3 Faculty of Biomedical Engineering, Czech Technical University in Prague, nam. Sitna 3105, 272 01 Kladno, Czech Republic; 4 Institute of Macromolecular Chemistry of the CAS, Heyrovskeho nam. 2, 162 06 Prague 6 Prague, Czech Republic; 5 University of Chemistry and Technology, Prague, Technicka 5, 166 28 Prague 6 Prague, Czech Republic; 6 ARC Centre of Excellence for Nanoscale BioPhotonics, School of Science, RMIT University, Melbourne, VIC 3001 Australia; 7 Nuclear Physics Institute of the CAS, 250 68 Husinec-Rez 130 Prague, Czech Republic

## Abstract

Energetic ions represent an important tool for the creation of controlled structural defects in solid nanomaterials. However, the current preparative irradiation techniques in accelerators show significant limitations in scaling-up, because only very thin layers of nanoparticles can be efficiently and homogeneously irradiated. Here, we show an easily scalable method for rapid irradiation of nanomaterials by light ions formed homogeneously in situ by a nuclear reaction. The target nanoparticles are embedded in B_2_O_3_ and placed in a neutron flux. Neutrons captured by ^10^B generate an isotropic flux of energetic α particles and ^7^Li^+^ ions that uniformly irradiates the surrounding nanoparticles. We produced 70 g of fluorescent nanodiamonds in an approximately 30-minute irradiation session, as well as fluorescent silicon carbide nanoparticles. Our method thus increased current preparative yields by a factor of 10^2^–10^3^. We envision that our technique will increase the production of ion-irradiated nanoparticles, facilitating their use in various applications.

## Abstract (web summary)

Mass production of nanoparticles containing well-controlled structural defects is a challenge. Here the authors demonstrate the feasibility of homogeneous ion irradiation generated in a nuclear reactor, for the preparation of fluorescent nanodiamonds and silicon carbide nanoparticles.

## Introduction

Over the past two decades, nanomaterials research has generated a wealth of experimental and theoretical data showing that the atomic structure and morphology of nanomaterials can be changed in a controllable manner^1,2^. While a chemical approach enables a plethora of synthetic modifications to the nanomaterial surface, currently available tools for post-preparative tailoring of the inner atomic structure of nanoparticles are based mostly on use of ionizing radiation and thermal annealing.

Among ionizing particles, energetic ions are attractive for materials science because they are very efficient in causing controlled structural defects in solid materials. Modification with energetic ions thus represents a key approach to the creation of a variety of functional nanostructured materials, which has enabled advances in numerous research fields. In optics, this approach is used to create lattice point defects, including vacancies, color centers^3,4^, and single-photon emitters^5^. In nanoscience, researchers have used modification with energetic ions to fabricate and tailor new types of materials^1,2,6,7^, including magnetic^8^, semiconductor^9^, and carbon^10^ nanomaterials. Irradiation with energetic ions also provides a means to radiolabel nanoparticles for biological tracing^11,12^.

Most nanoparticles can be engineered using so-called light ions [^1^H^+^, ^2^H^+^, ^3^He^+^, ^4^He^+^, α particles (^4^He^2+^), and ^7^Li^+^]. Compared to heavier ions, their range in materials is much higher^2^ and they cause less damage^13^. For example, irradiation with α particles or He^+^ ions has been used for tuning of the optical, electric, and magnetic properties of various nanomaterials—including graphene^14^, carbon^15^, and boron nitride^13^ nanotubes; semiconductors;^16^ magnetic nanoparticles;^17^ silica;^4^ and polymers^18^.

Although there is an impressive range of suggested applications for ion-irradiated nanoparticles, a major challenge remains in well-controlled mass preparation of these particles. To achieve uniform irradiation of a sufficient amount of material in accelerator sources, the ion beam is defocused and collimated just before entry to the target. However, the ion density may vary up to 20% due to distribution of ion density in the beam cross section. Due to beam energy dispersion, the ion ranges in the target also may differ significantly^19^. For energetic ions, penetration depth is typically millimeters to centimeters in the case of p^+^, but only micrometers to hundreds of micrometers for heavier ions, depending on ion energy and target density. The energetic ions lose energy along their path and end their way with the Bragg peak, beyond which there is negligible influence on the matter. Moreover, nanoparticle powders exhibit poor thermal conductivity and may overheat under irradiation, necessitating the use of thin nanoparticle layers in the target to prevent thermally induced alterations of the nanoparticles^20^. These reasons make scale-up of production extremely challenging, because only very thin layers of nanoparticles can be efficiently and homogeneously irradiated^3,21^.

Here, we describe an approach for mass production of ion-irradiated nanoparticles using light ions (α particles and ^7^Li^+^ ions) generated in situ. The target nanoparticles are dispersed in boron(III) oxide (which can be ^10^B-isotopically enriched) and placed in an isotropic neutron flux, where a neutron-induced reaction on ^10^B occurs homogeneously. Our approach utilizes the advantages of neutrons, including their long penetration depth into the target determined mainly by their absorption cross sections, the absence of threshold energy for a nuclear reaction, and the availability of scale-up of irradiated material to tens of grams. The all-directional local flux of light ions formed in situ from ^10^B uniformly irradiates the surrounding nanoparticles. Moreover, using high neutron fluence rates (10^12^–10^14^cm^–2^ s^–1^), which are routinely available in experimental nuclear reactors, we also achieve unusually high fluxes of energetic ions. Therefore, we gain comparable effects to hours of irradiation in accelerator devices in a few minutes and with much larger volumes. We demonstrate the benefits of our approach for the production of two fluorescent nanomaterials: diamond nanocrystals bearing fluorescent nitrogen-vacancy (NV) color centers in the crystal lattice (FNDs) and cubic silicon carbide nanoparticles bearing carbon antisite-vacancy pairs. Both nanomaterials are currently of great research interest because they provide unprecedented optical, electronic, and magnetic properties.

## Results

### Creation of NV centers in nanodiamonds

NV centers have been thoroughly studied for their unique applications as ultrasensitive magnetic^22–24^ and electric^25^ field sensors, single-photon emitters^26^, and chemical probes^27–29^. The fluorescence of NV centers is spin-dependent, which enables coherent manipulation of single NVs^30^ and measurement of optically detected magnetic resonance of single spins in ambient conditions^31^. FNDs show low toxicity, and their use as bright near-infrared fluorescent probes in high-resolution imaging^32–34^ and nanomedicine^35–38^ recently has been demonstrated^39^. Despite recent advancements in preparation procedures of FNDs, the currently available techniques involve primarily time-consuming and expensive irradiation with energetic ions^3,19,21,40–42^ or electrons^42–44^. Current irradiation approaches are summarized in recent reviews^45,46^.

**Fig. 1:**
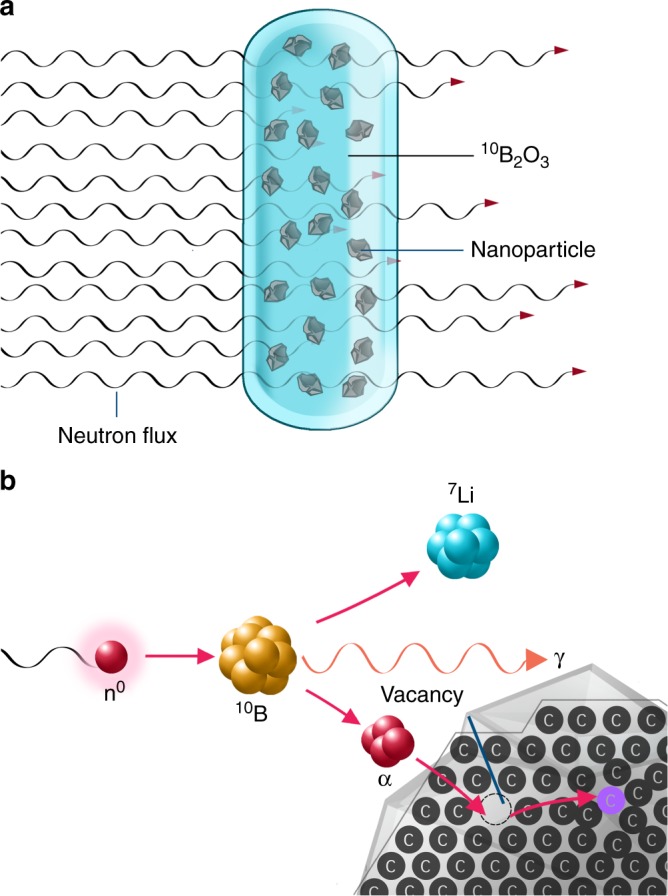
Basic principle of the implantation of energetic ions generated in situ into nanoparticles. **a** A container containing nanoparticles embedded in a glassy melt of ^10^B_2_O_3_ exposed to a neutron flux. **b** Detail of α particles and ^7^Li^+^ ions formed in situ by ^10^B neutron capture entering a nanodiamond particle and creating vacancies inside

The formation of NV centers in NDs is technically a two-step process and typically involves generation of vacancies in the diamond lattice using irradiation with energetic particles followed by recombination of vacancies with atomic nitrogen impurities upon high temperature annealing^3,19,40–42^. To efficiently produce energetic light ions (α particles and ^7^Li^+^ ions) creating the vacancies, we used capture of neutrons by ^10^B (Fig. 1)^47^. Two reaction channels exist, described by equations (1) and (2), with different probabilities (*P*)

1 $${}_5^{10}{\mathrm{B}} + {}_0^1n = {}_2^4{\mathrm{He}} + {}_3^7{\mathrm{Li}} + \gamma \left( {0.48\,{\mathrm{MeV}}} \right) + 2.31\,{\mathrm{MeV}}$$

*P* = 94%

2 $${}_5^{10}{\mathrm{B}} + {}_0^1n = {}_2^4{\mathrm{He}} + {}_3^7{\mathrm{Li}} + 2.79\,{\mathrm{MeV}}$$

*P* = 6% with a total absorption cross section of ~3800 barns for thermal neutrons^48^. We utilized these nuclear reactions for isotropic irradiation of a bulk sample containing homogeneously distributed nanoparticles, instead of exposing a thin layer of nanoparticles to an energetic ion beam, the range of which is low and results in a characteristic non-homogeneous distribution of defects in material (Bragg peak). We generated energetic ions homogeneously in the entire sample volume by reaction of ^10^B with thermal neutrons (Fig. 1).

### Interaction statistics and damage rate of NDs

**Fig. 2:**
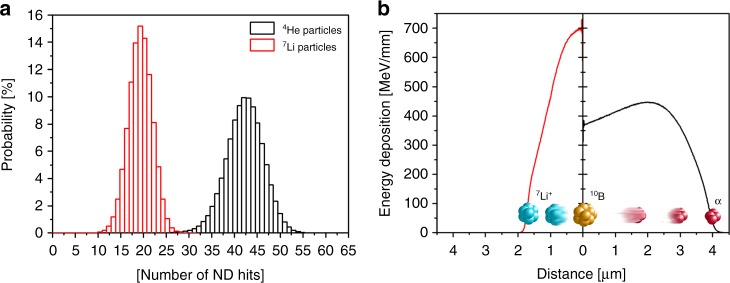
Interaction probability and energy deposition of α and ^7^Li^+^ particles. Compared to ^7^Li^+^ particles, α particles can interact with a higher number of nanodiamond (ND) particles and deposit energy over a larger distance in 33 weight % dispersion of 35-nm spherical NDs in ^10^B_2_O_3_. **a** Probability of the number of ND hits per one α particle and ^7^Li^+^ ion generated using reaction (1) and **b** energy deposition of these α particles (black) and ^7^Li^+^ ions (red) along their trajectory. On average, a single α particle with an energy of 1.47 MeV interacts along its ~3.9 μm trajectory with 42 nanoparticles before losing its kinetic energy. Similarly, a 0.84 MeV ^7^Li^+^ ion interacts along its ~1.7 μm trajectory with another 19 nanoparticles. The histograms and energy depositions were calculated from simulation of trajectories performed with the Geant4 toolkit

To understand the behavior of α particles and ^7^Li^+^ ions in a glassy melt, we first simulated their trajectories for a composite containing 33 weight % NDs (approximated with 35-nm diamond spheres) and 67% ^10^B_2_O_3_ (volume fraction of nanoparticles is 22.6%). We randomly distributed the NDs in the melt and using the Geant4 toolkit analyzed the trajectories of α particles and ^7^Li^+^ ions emitted from random surface points. From 2.5 × 10^6^ particle trajectories, we calculated the projected range (Supplementary Figure 1) and the average number of ND particles hit by one α particle or ^7^Li^+^ ion (Fig. 2a). Clearly, either an α particle or ^7^Li^+^ ion can penetrate far enough to create vacancies in dozens of individual NDs embedded in the ^10^B_2_O_3_ melt.

The vacancies are created with an efficiency that approximately corresponds to the amount of deposited energy from the ions. Fig. 2b shows these energy deposition curves in the ^10^B_2_O_3_ melt for an α particle and ^7^Li^+^ ion as a function of distance from a single ^10^B nucleus. Merging both depositions provides a region where we can anticipate active creation of vacancies, subsequently leading to formation of NV centers upon annealing. Note that in the case of less probable reaction (2), this region is slightly larger thanks to higher energy of both created ions (not shown). Because the nuclear reaction generating the α particles and ^7^Li^+^ ions occurs randomly in the entire volume of the melt, we expected the irradiation of nanoparticles to be very homogeneous and effective.

**Fig. 3:**
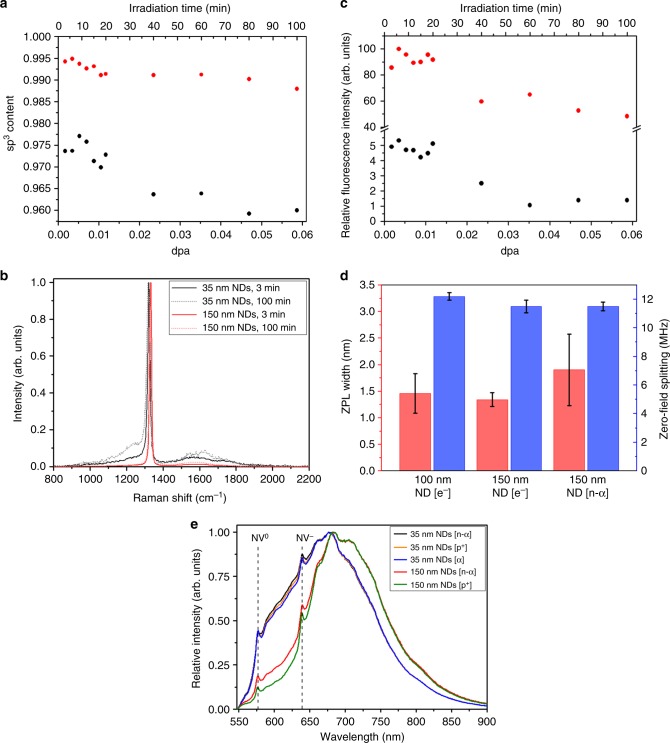
Spectral characterization of irradiated 35-nm and 150-nm nanodiamonds (NDs). **a**–**c** reveals how increased radiation damage **a**, **b** lowers relative fluorescence intensities **c** with increasing irradiation time in a nuclear reactor: **a** Content of sp^3^ carbon, **b** Raman spectra (for complete set of Raman spectra for all irradiation times, see Supplementary Figure 6), **c** Relative fluorescence intensity. **d** The average Gaussian NV^–^ ZPL linewidths^57^ measured at temperature of 4 K and the zero-magnetic-field splitting of the optically detected magnetic resonance (ODMR) dip for 150 nm ND [n-α], electron-irradiated NDs (150 nm ND [e^–^]), and electron-irradiated commercially available NDs (100 nm ND [e^–^]). The error bars indicate the standard deviation between values obtained for individual particles or particle aggregates (for spectra see Supplementary Figure 13). **e** Comparison of photoluminescence spectra of samples irradiated in a nuclear reactor [n-α] with samples irradiated in a cyclotron with protons [p^+^] or α particles [α]. For spectral measurements, the NDs were drop casted on a silicon wafer. The values of the *x*-axes in (**a**, **c**) are expressed as either irradiation time or the overall radiation damage expressed as dpa, which corresponds to the number of vacancies formed in NDs in our sample obtained from SRIM simulation (see Methods). The intensities in (**c**) are normalized to the diamond Raman band (the values are quantitatively comparable). The spectra in (**e**) are normalized at their maxima. Vertical dash lines labeled ZPL denotes the zero phonon line of the NV^−^ (wavelength 637 nm) and NV^0^ (wavelength 575 nm) color center.

The known composition and geometry of our sample, neutron fluence rates (see Methods) and involvement of self-shielding of thermal neutrons by ^10^B allowed us to calculate the effective thermal neutron fluence rate inside the melt^49^ and the corresponding yields of ions. Irradiation of our sample yielded 5.34 × 10^13^ s^−1^ of either α particles or ^7^Li^+^ ions (1.07 × 10^14^ s^−1^ in total). Based on these values, we calculated the number of created vacancies in NDs for each type of energetic ion using the SRIM simulation package^50^. The obtained values of 1.60 × 10^15^ and 4.07 × 10^15^ s^−1^ for α particles and ^7^Li^+^ ions, respectively, indicate effective and rapid formation of vacancies in the NDs present in the neutron-irradiated melt. The known composition of our sample enabled us to calculate an overall damage rate of 9.78 × 10^–6^ dpa s^−1^ for NDs [which was used for recalculation of irradiation times to overall radiation damage as displacement per atom (dpa), see Fig. 3a, c].

### Irradiation of samples in a nuclear reactor

To reach the highest efficacy of the irradiation procedure, it is essential to keep the nanoparticles in close contact with the ^10^B-rich environment. We met this condition by creating a dispersion of the nanoparticles in molten ^10^B_2_O_3_, which is formed by thermal dehydration of boric acid (H_3_^10^BO_3_). As a primary source of ^10^B, we used isotopically enriched boric acid (99.5 mole % ^10^B), because boric acid with natural isotopic abundance contains only 20 mole % ^10^B and 80 mole % inert nuclide ^11^B. Notably, H_3_^10^BO_3_ is generally available and inexpensive because large amounts are produced for the nuclear industry.

We prepared two glass melts containing a 33% dispersion of 35-nm and 150-nm NDs in ^10^B_2_O_3_. Their scanning electron microscopy (SEM) micrographs indicated homogeneously dispersed nanoparticles without signs of major aggregation or separation of both components (Supplementary Figure 2). The size of the visible granules roughly corresponds to the size of the nanoparticles.

To generate all-directional local flux of α particles and ^7^Li^+^ ions creating vacancies in ND crystals, we loaded both glass melts in quartz tubes and irradiated them in a nuclear reactor for various times ranging from 3 to 100 min. After irradiation, we dissolved the ^10^B_2_O_3_ matrix in NaOH solution and further processed the NDs in a similar manner as previously established for cyclotron-irradiated samples (annealing to form fluorescent NV centers and oxidation by air followed by treatment with a mixture of mineral acids)^19,33,51^. Isolation from the ^10^B_2_O_3_ melt was almost quantitative in yield and provided NDs with the characteristic size distribution (Supplementary Figure 3) and colloidal stability in aqueous solutions (Supplementary Figure 4). Zeta potentials were −46.7 mV for 35-nm NDs and −41.0 mV for 150-nm NDs, suggesting strong Coulombic stabilization by negative charge of deprotonated carboxylates created by oxidation on the surface of the nanoparticles.

To optimize the irradiation time, we next aimed to estimate the minimum required dose for obtaining highly bright NDs. To our surprise, we found that it was possible to reduce the dose to 1.76 × 10^−3^ dpa (corresponds to 3 min—the shortest time for which irradiation is reproducible, due to loading-unloading lags into the water-cooled channels of LVR-15 nuclear reactor we used), while maintaining the quality and the intensity of ND fluorescence (Fig. 3c). In contrast, the control samples of NDs irradiated with neutrons only (without the presence of ^10^B_2_O_3_) show more than one order of magnitude lower fluorescence intensity (Supplementary Figure 5). This indicates that the structural effects on NDs originate predominantly from interaction with α particles and ^7^Li^+^ ions, but not directly from interaction with neutrons.

### Irradiation damage and other properties of NDs

Using Raman spectroscopy, we analyzed the diamond lattice irradiation damage for both types of NDs. In general, we observed a slight decrease in sp^3^ and increase in sp^2^ carbon content caused by crystal lattice damage with increasing irradiation time (Fig. 3a, b). The higher amount of sp^2^ carbons for smaller NDs can be explained by their higher surface/volume ratio, which is consistent with the observation that the formation of sp^2^ phases in NDs occurs preferentially in the surface region^52^. Consistently, we observed the highest fluorescence intensity for NDs irradiated for less than 20 min (Fig. 3c). With longer irradiation times, the yield of NV centers after annealing gradually drops, which correlates with the observed progressive crystal lattice degradation into sp^2^ and amorphous structures (Fig. 3a). In addition to the G-band intensity (~1600 cm^−1^), we monitored the appearance of a peak at ~1230 cm^−1^ (Fig. 3b and Supplementary Figure 6), which appears in Raman spectra of ion-irradiated diamond^53^ due to presence of extended defects such amorphous carbon inclusions. Correspondingly, the progressive crystal lattice degradation occurring at longer irradiation times (≥ 15 min, ≥ 8.81 × 10^−3^ dpa; Fig. 3a) is clearly reflected in the correlation between the intensities of the G-band and the ~1230 cm^−1^ peak (Supplementary Figure 8A). Low level of damage caused by irradiation under optimized conditions is demonstrated by the fact that the intensities of the ~1230 cm^−1^ peak, as well as the G-band were undistinguishable for non-irradiated NDs and NDs subjected to low irradiation times ( ≤ 12 min, which corresponds to  ≤ 7.04 × 10^−3^ dpa; Supplementary Figure 8B).

Even though Raman spectroscopy is the method of first choice to evaluate defects in carbon materials, it might not be sensitive enough to reveal local defects and strains in the diamond lattice that could have a negative impact on optical properties of nearby NV centers and limit their future applications. We have performed low-temperature confocal fluorescence spectroscopy to evaluate the quality of the NV centers in NDs irradiated under optimized conditions. It is known that the linewidth of NV^–^ ZPL broadens with increasing local lattice damage^54,55^ and other sources of local strain, such as impurities^56–58^. We have measured the NV^–^ ZPL linewidth at the temperature of 4 K. For comparison with 150 nm ND [n-α], we chose electron-irradiated NDs (150 nm ND [e^–^]) and commercially available, electron-irradiated NDs (100 nm ND [e^–^]) (see Methods for details). Fig. 3d shows the average Gaussian NV^–^ ZPL linewidths^57^ (see Supplementary Figure 12 and 13 for confocal images and the spectra, respectively). The average linewidth of the neutron-irradiated particles was statistically not significantly different from the electron-irradiated samples.

Another measure of the local strain in the diamond lattice is the splitting of the optically detected magnetic resonance (ODMR) signal at zero external magnetic field^56^. We find this zero-field splitting to be between 11.5 MHz and 12.2 MHz at room temperature for all samples (Fig. 3d), indicating that a comparable average local strain is present in all samples, irrespective of the irradiation method (see Supplementary Figure 14 for ODMR spectra). These values are in the range reported even for non-irradiated NDs (11.6 MHz to 13.0 MHz)^59^. Furthermore, in a separate study of the optical properties of NDs, we found the spin relaxation times T_1_ of this set of particles do not differ (unpublished results). Since the T_1_ spin relaxation time is also known to be sensitive to local lattice damage^56^, our measurements suggest that the spin and fluorescent properties of NDs [n-α] produced here are overall of high quality and they are comparable with the commonly used electron irradiation.

### Comparison with other irradiation methods

Next, we compared our results with data on the creation of NV centers in bulk diamond crystals irradiated only by fast neutrons (without the presence of ^10^B_2_O_3_)^60^. In these cases, the number of created NV centers grew linearly with neutron fluence, reaching an optimum at 7 × 10^17^ cm^−2^ and dropping rapidly above this value. Notably, under the conditions used in our setup, the estimated optimum dose would correspond to ~27 h of fast neutron irradiation, which is three orders of magnitude longer than the 3 min achieved with our approach. Correspondingly, 50 h of neutron irradiation^61^ with a fluence of 5 × 10^17^ cm^−2^ and tens of hours^62^ with neutron fluencies ~10^17^ were necessary for effective creation of NV centers in bulk diamond crystals. Inelastic fast neutron scattering causes the vacancy formation in samples without the presence of ^10^B_2_O_3_, while thermal neutron capture by ^10^B leads to creation of vacancies upon interactions of the formed α particle and ^7^Li^+^ ions with carbon atoms. To ascertain the dominant process contributing to NV center formation in our sample, we compared the cross sections of inelastic neutron scattering on diamond carbon atoms (for fast neutrons it is ~4.0 barns, for thermal neutrons 4.75 barns)^47^ and thermal neutron capture by ^10^B (3800 barns)^48^, and found a difference of approximately three orders of magnitude. Although these cross sections relate to different processes (direct vs. indirect interaction of neutrons with carbon atoms), the vast difference between them corresponds well with the observed predominance of neutron capture by ^10^B in vacancy formation (Supplementary Figure 5).

Moreover, FNDs produced in this way show spectral features consistent with results obtained previously in an accelerator^41^. Specifically, the 150-nm NDs had much higher fluorescence intensity than the smaller 35-nm NDs (Fig. 3c). Because the maximum vacancy concentration is an increasing function of the particle size^63^, the vacancy capture efficiency strongly rises with increasing size of the diamond crystal. The migration path of the vacancies to the surface of nanoparticles during annealing is shorter for smaller particles, and their recombination with nitrogen atoms is less effective.

The NV^–^/NV^0^ zero phonon line (ZPL) intensity ratios and the width of the ZPL and phonon replicas were similar for neutron-irradiated NDs in ^10^B_2_O_3_ glass and for FNDs we prepared using direct cyclotron irradiation with either p^+^ or α particles (Fig. 3e). Because these spectral parameters are related to the crystal properties (formed irregularities, other defect centers) and surface properties^64,65^, the similarities in the observed spectra indicate that the samples prepared by the different types of irradiation have comparable damage to the crystal lattice.

Although the spectral shapes and overall fluorescence of our FNDs were very similar to those of FNDs irradiated in an accelerator, we were interested in whether our procedure increases the homogeneity of irradiation, i.e., whether the material contains a higher fraction of FNDs. To distinguish the fluorescent and non-fluorescent NDs present in a large ensemble at the single-particle level^19,66^, we utilized simultaneous measurement of fluorescence-lifetime imaging microscopy (FLIM) and atomic force microscopy (AFM) (Supplementary Figure 7). We found that the fraction of FNDs in material irradiated in a nuclear reactor increased by a factor of 2.6 compared to optimally^41^ p^+^-irradiated pellet target with NDs in an accelerator (49% vs. 19%). Moreover, the particles irradiated in a nuclear reactor were brighter on average and contained a significantly higher fraction of very bright particles (fluorescence intensity corresponding to ~5 NV centers and higher). However, the largest fraction of particles exhibited lower fluorescence (corresponding to < 3 NV centers); see histograms in Supplementary Figure 9a, b.

This increase in the fraction of fluorescent particles is close to the enhancement factor of 3.2 achieved for a homogeneous liquid target containing a colloidal aqueous solution of NDs compared to an optimized pellet target^19^. Note that both types of accelerator irradiation took 4.5 h (compared to 3 min in a reactor).

### Irradiation of silicon carbide nanoparticles

To demonstrate the capability of our method to produce lattice defects in a different material, we focused on silicon carbide (SiC), one of the key materials used in development of next-generation photonic and electronic devices^5^. SiC forms various polytypes harboring a range of different lattice defects^67^, which can be created upon irradiation with energetic particles. Some exhibit single-photon emission and can be employed as room-temperature solid-state “qubits”^68,69^. We used the cubic 3C-SiC polytype, enabling creation of a single-photon emitter—the carbon antisite-vacancy pair^70^. In addition, 3C-SiC nanoparticles are biocompatible and can be used as photostable fluorescent labels in cell imaging^71–73^. As with FNDs, wider use of photoluminescent SiC is limited because the current irradiation approaches allow production of only a small amount of material.

**Fig. 4:**
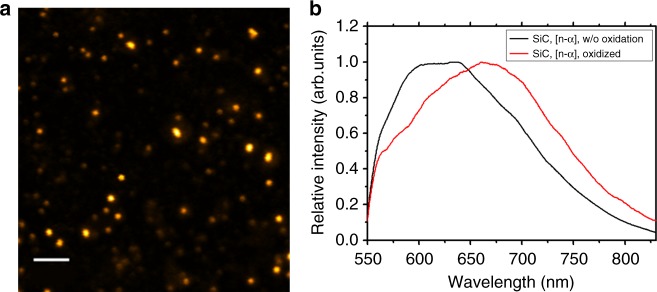
Spectral characterization of irradiated SiC nanoparticles. **a** Confocal image of irradiated, annealed and oxidized SiC nanoparticles deposited on a glass cover slip. The scale bar corresponds to 5 μm. **b** Photoluminescence spectrum of only irradiated (w/o oxidation) and of irradiated, oxidized and annealed (oxidized) SiC nanoparticles. The band around 670 nm was assigned to the carbon antisite-vacancy pair^70^

Similarly as for NDs, we prepared a composite of cubic 3C-SiC nanoparticles with B_2_O_3_, irradiated it in a nuclear reactor, and processed the sample (see Methods). Using the SRIM simulation package^50^, we calculated the number of created vacancies in SiC as 1.41 × 10^15^ and 3.587 × 10^15^ s^−1^ for α particles and ^7^Li^+^ ions, respectively, indicating a slightly higher overall damage rate of SiC (1.42 × 10^–5^ dpa s^−1^) compared to ND. After irradiation for 15 min (1.28 × 10^–2^ dpa) and subsequent annealing and oxidation in air, we obtained strongly luminescent nanoparticles (Fig. 4a) with one dominant peak around 670 nm in the photoluminescence spectrum (Fig. 4b). This band was previously observed in electron-irradiated and oxidized 3C-SiC nanoparticles and was assigned to the carbon antisite-vacancy defect^70^.

In nanoparticles that were only irradiated (without subsequent annealing and oxidation), another band appeared around 600–650 nm. The dominance of one spectral feature did not occur in samples irradiated for shorter time (3 min, 2.56 × 10^–3^ dpa) nor in non-irradiated, annealed and oxidized samples. Consistent with previous findings, we observed only luminescence features with varying spectral position between 600–800 nm (Supplementary Figure 10) as common for intrinsic defects in this form of SiC^74,75^. Oxidation further improved the solubility of both irradiated and non-irradiated samples and led to stabilization of luminescence. We observed bleaching of the non-oxidized samples, while oxidized nanoparticles did not bleach over tens of minutes of measurements. Overall, our data point towards effective creation of photoluminescent point defects in cubic SiC nanoparticles and demonstrate the capability of our irradiation method for nanomaterials other than NDs.

### Scale-up of the method and mass production of FNDs

Finally, we applied our method to the preparation of a large amount of FNDs. First, we adjusted the preparation, irradiation and processing procedures for large-scale conditions (oxidation of ~160 g NDs, ~340 g of melting mixture, liters of solutions). Based on the extremely short durations needed for effective irradiation of ND-^10^B_2_O_3_ composite, we expected that the process could yield enough vacancies using less expensive B_2_O_3_ with natural isotopic abundance (20 mole % ^10^B). We thus used B_2_O_3_ rather than ^10^B-isotopically enriched H_3_^10^BO_3_, which also facilitated the melting procedure (no hot steam evolved upon H_3_BO_3_ thermal dehydration). For irradiation, we designed double-walled containers enabling sufficient heat dissipation to cooling media in the nuclear reactor and isotropic irradiation of the composite by neutrons (Supplementary Figure 11C). Upon irradiation of 10 containers containing a total of 240 g ND-B_2_O_3_ composite (each container for 3 min, total irradiation time 0.5 h) and standard processing, we obtained 70 g FNDs (95% yield based on the initial amount of the composite). To the best of our knowledge, this amount is more than two orders of magnitude higher than any reported FND preparation.

The FNDs showed equal spectral features compared to our initial small batches irradiated in capillaries (Supplementary Figure 11A, B). The intensities of the ~1230 cm^−1^ peak and the G-band in Raman spectra were undistinguishable for the non-irradiated NDs and for the small batch irradiation. The large-scale procedure thus provided FNDs with high sp^3^ purity and a low level of lattice damage. Photoluminescence spectra confirmed the presence of NV centers in FNDs with NV^–^/NV^0^ ZPL intensity ratios identical to those of the corresponding small batch. Importantly, the scale-up procedure only slightly affected the homogeneity of irradiation and FND brightness (Table 1 and Supplementary Figure 9c), as documented by simultaneous FLIM and AFM measurements. The fraction of FNDs in the material was higher by a factor of 2.2 than optimally p^+^-irradiated pellet target with NDs in an accelerator (41% vs. 19%). These results show that the large-scale preparation provides FNDs with comparable quality to the small scale.

**Table 1:** Fraction of fluorescent particles and fluorescence intensities for different irradiations

	Irradiated in reactor	Proton irradiated	Irradiated in reactor (large scale)
Fraction of fluorescent particles	49%	19%	41%
Normalized average fluorescence intensity per particle (arb. units)	3.2	1.7	2.7
Normalized median fluorescence intensity per particle (arb. units)	2.1	1.4	1.8

The table compares fluorescent nanodiamonds (NDs) prepared by neutron irradiation in a reactor and proton irradiation in a cyclotron using an optimized pellet target with large-scale production of NDs in the reactor (irradiation of 240 g ND-B_2_O_3_ composite). The fluorescence intensity is normalized to the average intensity of one NV center in a ND particle. Parameters were calculated from more than 400 particles. Isotropic irradiation with energetic light ions leads to a higher fraction of fluorescent particles and to higher fluorescence intensities.

## Discussion

We described an easily scalable method for production of light-ion-irradiated nanoparticles utilizing α particle and ^7^Li^+^ ions generated in situ. The target nanoparticles embedded in ^10^B-isotopically enriched boric oxide are placed in a neutron flux, where neutron-induced nuclear reaction on ^10^B occurs homogeneously, producing an isotropic flux of light ions. Our method thus combines the advantages of the neutron and ion irradiation approaches. We demonstrated its usefulness for production of FNDs bearing nitrogen-vacancy color centers and of 3C-SiC nanoparticles with carbon antisite-vacancy pairs. In a large-scale pilot experiment, we prepared 70 g FNDs during a half-hour irradiation session using boric oxide with natural isotopic abundance of ^10^B. The irradiation was highly homogeneous, producing material with a high fraction of bright fluorescent particles. Because of the favorably high cross section of ^10^B for neutron capture, our method can operate with extremely rapid irradiation times of only a few minutes per one batch. Overall, our large-scale experiment demonstrated that short irradiation time and space capacity of irradiation channels in the nuclear reactor provides the possibility of semi-continuous production of hundreds of grams of FNDs per day. In comparison to irradiation with electrons^43^ or energetic ions in accelerators^3,19,41,42^, in which the yield typically reaches hundreds of milligrams per day, we increased the current production rates of ion-irradiated nanoparticles by a factor of ~10^2^–10^3^. This allows for unprecedentedly high preparative yields and economically feasible production of irradiated nanoparticles (the price per hour of irradiation is similar for nuclear reactors and accelerators).

We envision that our technique, combined with general accessibility to nuclear reactors (currently, 59 research nuclear reactors useful for this type of irradiation with public access are operating worldwide)^76^, can facilitate production of well-defined light-ion-irradiated nanoparticles that can be widely used in diverse applications, such as in semiconductor, magnetic, quantum sensing, optical, and bioimaging devices.

## Methods

### Chemicals

Sodium hydroxide, hydrochloric acid (35%), nitric acid (65%), and sulfuric acid (96%) were purchased from Penta (Czech Republic). Potassium nitrate and hydrofluoric acid (40%) were purchased from Sigma Aldrich (Prague, Czech Republic). All chemicals were p.a. quality and were used as received without further purification. Boric acid enriched to 99.5% ^10^B was supplied by Katchem Ltd., Czech Republic. Boron (III) oxide (99.9+ %) was purchased from Strem Chemicals, Inc. Deionized water used for all washing steps and preparation of solutions was prepared with a Millipore Synergy UV Ultrapure water system. The sample of commercially available electron-irradiated fluorescent NDs (high brightness) was obtained from Adamas Nanotechnologies, USA (abbreviated as 100 nm ND [e^–^]).

### ND and SiC pretreatment

NDs were supplied by Microdiamant Switzerland (MSY 0–0.05 and MSY 0–0.25, containing ~100–200 ppm of natural nitrogen impurities). The NDs were oxidized by air in a furnace (Thermolyne 21100 tube) at 510 °C for 5 h and subsequently carefully purified to remove trace amounts of elements (e.g., iron) that may activate in neutron flux, producing undesirable radioactive contamination of the product. The product had negligible radioactivity after the following purification was implemented. The NDs were treated with a mixture of H_2_SO_4_ and HNO_3_ (9:1) at 90 °C for 3 days and washed with water, 1 M NaOH, and 1 M HCl. They were washed an additional 5 times with water and then freeze-dried. Purified ND powder (500 mg) was mixed with 2.0 g H_3_^10^BO_3_ ground in a mortar and transferred into a synthetic corundum crucible. The mixture was placed in a vertical furnace (Thermolyne 21100 tube) and heated to 600 °C for 5 min (until the development of water vapor ceased). The temperature was then increased to 700 °C, and the melt was homogenized by mixing and left to cool to RT. The final glassy composite was first ground in a mortar and then pulverized in a small ball mill. The typical weight loss within such melting was 39% due to dehydration of boric acid to boron(III) oxide. The final melt used for irradiations contained 33 weight % NDs and 22% ^10^B.

Cubic SiC nanoparticles (PlasmaChem GmbH, PL-CT-SiC, 150–200 nm; 1.00 g) were mixed with boron oxide (2.25 g) and ground in an agate mortar. The mixture was transferred into a porcelain crucible and heated in a vertical furnace (Nabertherm RT 50–250/13) at 720 °C for 10 min. The viscous melt was homogenized by mixing, scraped out with a spatula, and left to cool down to RT. The final light-gray glassy composite was ground in a ball mill and finely pulverized in a mortar.

### Irradiation in nuclear reactor and treatment of ND and SiC

The powderized melts of ND and ^10^B_2_O_3_ were sealed in quartz glass, sodium-free capillaries (inner diameter, 1.5 mm; height of the melt, 11 mm), inserted in an aluminum container, and irradiated in a vertical water-cooled (~45 °C) channel H8 positioned in the Be reflector of the LVR-15 nuclear reactor of Research Centre Rez, Ltd. at neutron fluence rates of 2 × 10^13^ cm^−2^ s^−1^, 1 × 10^13^ cm^−2^ s^−1^, and 7 × 10^12^ cm^−2^ s^−1^ for thermal, epithermal, and fast neutrons, respectively, for various periods of time (3–100 min). Neither NDs nor quartz glass should contain traces of sodium because the natural monoisotope ^23^Na is readily neutron-activated into ^24^Na, which is a beta and gamma emitter with a half-life of 14.997 h. If there are traces of sodium in the starting materials, an easy option is to allow ^24^Na to decay for 10 half-lives (one week, or a shorter time if less contaminated) after irradiation. The sample can be handled then as non-radioactive. After irradiation, the capillaries were opened and left overnight in a vial with 6 M NaOH at 60 °C to dissolve boron(III) oxide. The residue adhering to quartz glass was released in an ultrasonic bath. Supernatant was washed gradually with 6 M NaOH, H_2_O, 1 M HCl, and 5 times with H_2_O. Possible quartz glass splinters were separated by sedimentation. The supernatant was treated with concentrated HF for 12 h; washed with H_2_O, 1 M HCl, and 5 times with H_2_O; and lyophilized.

Six quartz glass capillaries, each filled with 55 mg of SiC-B_2_O_3_ composite, were sealed into aluminum containers. The capillaries were irradiated for various periods of time (3–15 min) in vertical water-cooled (~45 °C) channel H8 of the LVR-15 nuclear reactor at neutron fluence rates of 3.6 × 10^13^, 8.4 × 10^12^ and 5.6 × 10^12^ cm^−2^ s^−1^ for thermal, epithermal and fast neutrons, respectively. After irradiation, the capillaries were opened and twice washed with 2 M NaOH at 95 °C to dissolve boron oxide. The supernatant was gradually washed with H_2_O (twice), 1 M HCl, and H_2_O (five times) and lyophilized. The obtained SiC powder was oxidized by air in a furnace (Nabertherm RT 50–250/13) for 3 h at 550 °C.

### Large-scale irradiation of NDs

For large-scale preparation, NDs were supplied by Henan Huifeng Diamond Co., Ltd., China (HFD-F, 35 nm). A thin layer of NDs (166.8 g) in a ceramic dish was oxidized by air in a furnace (Clasic CZ, 1013 S) at 510 °C for 3 h, providing 119.8 g of oxidized NDs (72% yield). The oxidized NDs (105.0 g) were mixed with boron oxide (236.3 g), ground in a mortar, and heated in 20 g doses in a vertical furnace (Nabertherm RT 50–250/13) at 700 °C, 10 min per dose. The viscous melt was homogenized by mixing, scraped out with a spatula, and left to cool down to RT with a 94% total yield (321.8 g). The final glassy composite was ground in a ball mill (Retsch MM 400) and finely pulverized in a mortar.

The 10 purpose-made aluminum containers (Supplementary Figure 11C) were filled with a composite powder (24 g per container), sealed and irradiated for 3 min in vertical water-cooled (~45 °C) channel H6 of the LVR-15 nuclear reactor at neutron fluence rates of 5.4 × 10^13^, 7.7 × 10^13^, and 6.5 × 10^12^ cm^−2^ s^−1^ for thermal, epithermal, and fast neutrons, respectively. After the irradiation, the containers were left for 2 weeks in a shielded hot cell to allow decay of residues of short-lived radionuclides created by neutron activation. The containers were opened and the obtained powder was mixed with 1000 ml of 10% NaOH, stirred at 95 °C for 1 h to dissolve boron oxide and possible aluminum residues and left to sediment overnight. The sediment was washed once with H_2_O, separated by centrifugation and treated with 800 ml of boiling *aqua regia* for 1 h to dissolve possible traces of long-lived radionuclides. The sediment was washed four times with H_2_O (Supplementary Figure 11D) and lyophilized. The final yield was 70 g of NDs in the form of a light gray powder (95% yield after workup; Supplementary Figure 11E).

### Irradiation with electrons

Purified ND powder (Microdiamant Switzerland, MSY 0–0.25) was irradiated in an external aluminum target holder for 21 h with a 16.6 MeV electron beam (1.25 × 10^19^ particles cm^−2^) extracted from MT-25 microtron, as previously described^29^. The sample is abbreviated as 150 nm ND [e^–^].

### Annealing and oxidation

All ND samples were annealed at 900 °C for 1 h in an argon atmosphere followed by air oxidation at 510 °C for 4 h at normal pressure in a Thermolyne 21100 tube furnace calibrated with an external thermocouple (Testo AG 1009). According to the transmission electron microscopy (TEM) image analysis^77^, the obtained particles were 35 nm in diameter.

### TEM measurement

For FND particle size distribution evaluation, we used image analysis of TEM micrographs (Supplementary Figure 3A, B). For each sample, we analyzed ~1000 particles, acquired their equivalent circular diameters, and recalculated them to volume-weighted histograms (Supplementary Figure 3C).

Samples for TEM were prepared similarly as described in our previous work^77^. Carbon-coated copper grids (Pyser) were oxidized in a UV-ozonizing chamber (UV/Ozone Pro Cleaner Plus, Bioforce Nanosciences) for 15 min, then incubated in poly(ethyleneimine) solution (MW = 2.5 kDa, 0.1 mg ml^−1^) for 10 min, washed with water, and incubated in an aqueous solution of NDs (0.1 mg ml^−1^) for 3 mins. Micrographs were taken with a JEOL JEM 1011 microscope at 80 kV acceleration voltage.

Analysis of particle size distributions was performed with ImageJ software using a previously described procedure^77^. Particle size was expressed as equivalent circular diameter (*d*_eq_), defined as the diameter of a circular particle with the same area as the particle of interest (*S*).

3 $$d_{{\mathrm{eq}}} = \sqrt {4S/\pi }$$

Equivalent diameters were used to calculate particle volume (PV).

4 $${\mathrm{PV}} = \frac{4}{3}\pi \left( {\frac{{d_{{\mathrm{eq}}}}}{2}} \right)^3$$

and subsequently for creation of volume-weighted histogram.

### SEM measurement

A silicon wafer (10 × 3 mm) with a small fragment of boron oxide-ND composite sample put on its surface was placed in a quartz tube. The sample was melted at 700 °C for 15 min under an argon atmosphere in a Thermolyne 21100 tube furnace and left to cool down to RT. Immediately after preparation, the silicon wafer with melted sample was fixed to a holder with double-sided tape and coated with a thin layer of gold. The morphologies of samples were observed by using a Hitachi S-4700 field emission scanning electron microscope (FE-SEM) at 15 kV.

### Geant4 and SRIM simulations

Geant4 v10.2 general particle transport toolkit^78,79^ was used to calculate the projected range and average number of ND particles hit by one α particle or ^7^Li^+^ ion in a dispersion of NDs in ^10^B_2_O_3_. The user application was developed using the TestEm11 extended electromagnetic example as a template.

ND particles were approximated by carbon spheres with radius 35 nm and density 3.5 g cm^−3^. ND particles were then randomly distributed into ^10^B_2_O_3_ material, with density 1.82 g cm^−3^, forming a homogeneous dispersion. Due to memory constraints, the nested replicated approach was applied to build the whole sample volume. The volume of the sample was filled by replicating a single building block with base 0.3 × 0.3 μm and height set to match the sample height. The sample volume was set to 2.7 × 2.7 × 6 μm for α particles and to 2.7 × 2.7 × 2.4 μm for ^7^Li^+^ ions. Primary particles generated by nuclear reaction (1) (1.47 MeV α particle and 0.84 MeV ^7^Li^+^ ion) were emitted perpendicular to the building block base plane, from a random point in a square with 100 nm sides placed in the center of the entry surface of the middle building block.

Two hundred and fifty different ND distributions inside the building block were generated, and for each of those configurations, calculations were run for 10^4^ primaries. Results are presented for the resulting 2.5 × 10^6^ primary particles for each type.

SRIM-2013 code (www.srim.org) was used to simulate damage of NDs and SiC upon irradiation. The damage rate (expressed as the number of vacancies created in the overall sample) was estimated using the known atomic composition (see ND and SiC pretreatment) and yields of 5.34 × 10^13^ s^−1^ for α particles (1.47 MeV) and ^7^Li^+^ ions (0.84 MeV) generated upon neutron irradiation in the overall sample. Because the range of the energetic ions in the material (a few μm) is negligible compared to the dimension of the sample (a cylinder of diameter 1.5 mm and height 11 mm), we considered all created ions to be captured in the sample. The dpa values were recalculated for the fraction of carbon (and silicon) atoms in the material.

### Raman and other measurements

The samples were prepared by drop-casting of the aqueous dispersion of NDs on the polished silicon wafer. Raman and luminescence spectra were measured using a Renishaw InVia Raman Microscope; the excitation wavelength was 514 nm (luminescence measurements) and 325 nm (Raman measurements) with 15 mW laser power, ×20 objective. The exposure time was 6 seconds, accumulation 10 times; 20 measurements were made on each sample. The Raman and luminescence spectra were taken at room temperature and normalized to the diamond Raman peak. The changes in sp^3^ carbon content were measured and evaluated according the literature procedure^80^. Raman spectra were analyzed using the Peak-o-mat program.

Low-temperature imaging and spectroscopy were performed using a custom-built confocal fluorescence microscope. Briefly, a 532 nm (60 µW) continuous-wave laser beam was focused onto the samples located inside a cryostat (Montana Instruments, Cryostation) through an optical window using a long working distance objective (Olympus, LC Plan N, ×50, NA 0.65). Fluorescence was collected with an avalanche photodiode (Excelitas, APD, SPCM-AQRH-14) for imaging and a spectrometer (Princeton Instruments, SpectraPro with a PIXIS CCD camera) to obtain fluorescence spectra. 532 nm laser line, dichroic and notch filters were used to separate excitation and fluorescence signals.

ODMR measurements (at room temperature): a modulated microwave field was created using a microwave signal generator (Rohde & Schwarz, SMIQ03B) with amplifier and delivered using a custom-built sample holder with an Ω-shaped wire. Excitation and PL detection was carried out as described above for low-temperature measurements, but using a higher numerical aperture objective (NA 0.9) and a lower excitation intensity (8 µW).

### FLIM and AFM measurements of ND

The FNDs were deposited on an oxygen-plasma-cleaned glass cover slip by dip coating for 5 min and rinsed using DI water (MilliQ). The concentration of the stock solution of nanoparticles was 0.001 mg ml^−1^. For FLIM, fluorescence images were taken using a time-resolved fluorescence confocal microscope (MicroTime200 – PicoQuant), with excitation wavelength 532 nm, 1.2 mW laser power, using a ×60 water-immersion objective (Olympus) and a 650 long pass filter (Edmund Optics, OD4). Data were processed using Matlab (R2014b, Mathworks). Selection of FNDs was performed using calculated fast fluorescence lifetime (FLIM) ( > 6 ns) and counts/pixel ( > 10 cts) thresholds. To obtain normalized PL intensity per particle, the measured fluorescence intensities were normalized to the calculated average fluorescence intensity of single NV center (based on correlation measurements). The normalized intensity therefore represents ~the number of NV centers in the particle. AFM images were taken on a JPK Nanowizard® AFM combined on the FLIM Microtime setup. Scans were performed using AC mode measurements using silicon probes (ACTA, with aluminum coating of the reflex side, ACTA300 – TL).

### Spectral characterization of SiC

Round microscope cover glasses with 25-mm diameter were used as substrates. The coverslips were immersed overnight in a concentrated KOH/methanol solution and then thoroughly washed with deionized water and dried. The samples were prepared by drop-casting 15 μL 0.01 mg ml^−1^ aqueous solution of SiC particles. To localize the particles, a time-resolved confocal fluorescence microscope system [MicroTime 200 (PicoQuant, GmbH) with ×60, 1.2 N.A. equipped with a 532 nm pulsed diode laser and water-immersion objective] was used to record fluorescence images. Fluorescence spectra of single particles were recorded in fixed-point measurement mode using a fiber-coupled Shamrock 303i spectrograph with an iXon Ultra EMCCD camera (Andor). Spectra were recorded at 30 μW excitation power and 15 s integration time. Final spectra were prepared by subtracting a dark spectrum recorded from an empty substrate under the same conditions.

## Electronic supplementary material

Supplementary Information

## Data Availability

Data available on request from the authors.
