# Supplementary material for: Extremely rapid isotropic irradiation of nanoparticles with ions generated in situ by a nuclear reaction
Source: Nat Commun. 2018 Oct 26;9:4467. doi: 10.1038/s41467-018-06789-8 (PMC6203839; doi:10.1038/s41467-018-06789-8)
Supplement: Supplementary file 1 — Supplementary Information [file 41467_2018_6789_MOESM1_ESM.pdf]

Extremely rapid isotropic irradiation of nanoparticles with ions generated *in situ* by a nuclear reaction

Martin Hruby and Petr Cigler – corresponding authors

Supplementary Figures

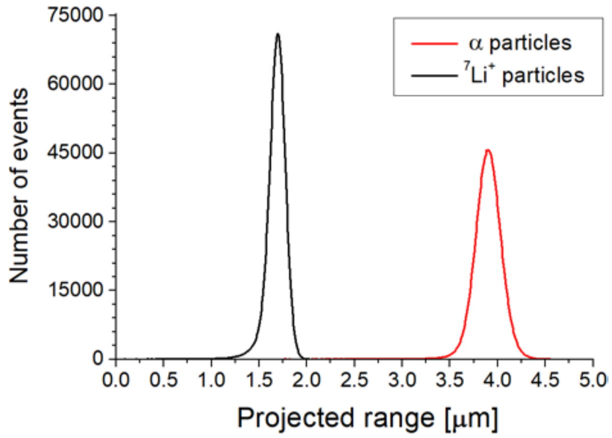

Supplementary Figure 1. Projected ranges for 1.47 MeV α particles and 0.84 MeV $^7Li^+$ particles. Simulated with Geant4 toolkit in a 33% dispersion of 35-nm NDs in $^{10}B_2O_3$.

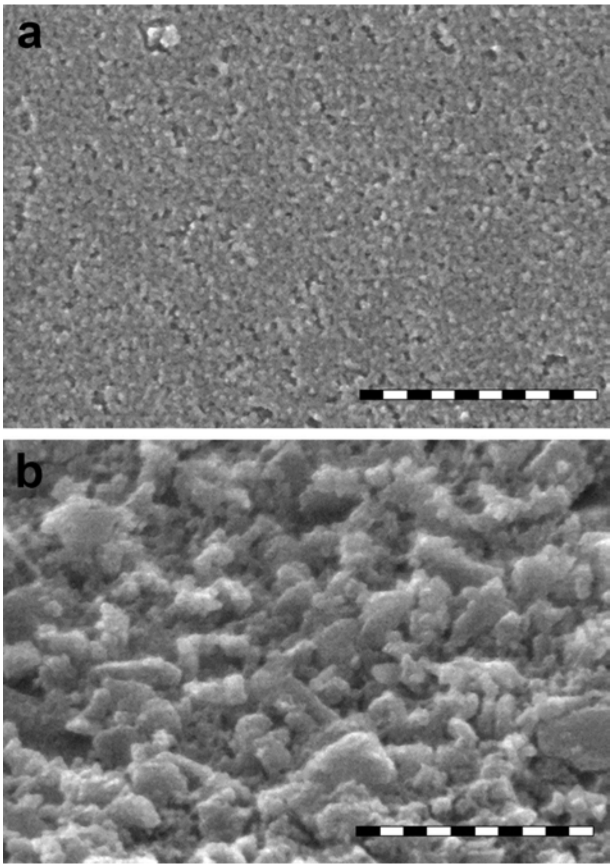

Supplementary Figure 2. SEM micrographs of $^{10}B_2O_3$ melts containing nanodiamonds (NDs). (**a**) 35-nm, (**b**) 150-nm NDs. The scale bars correspond to 500 nm.

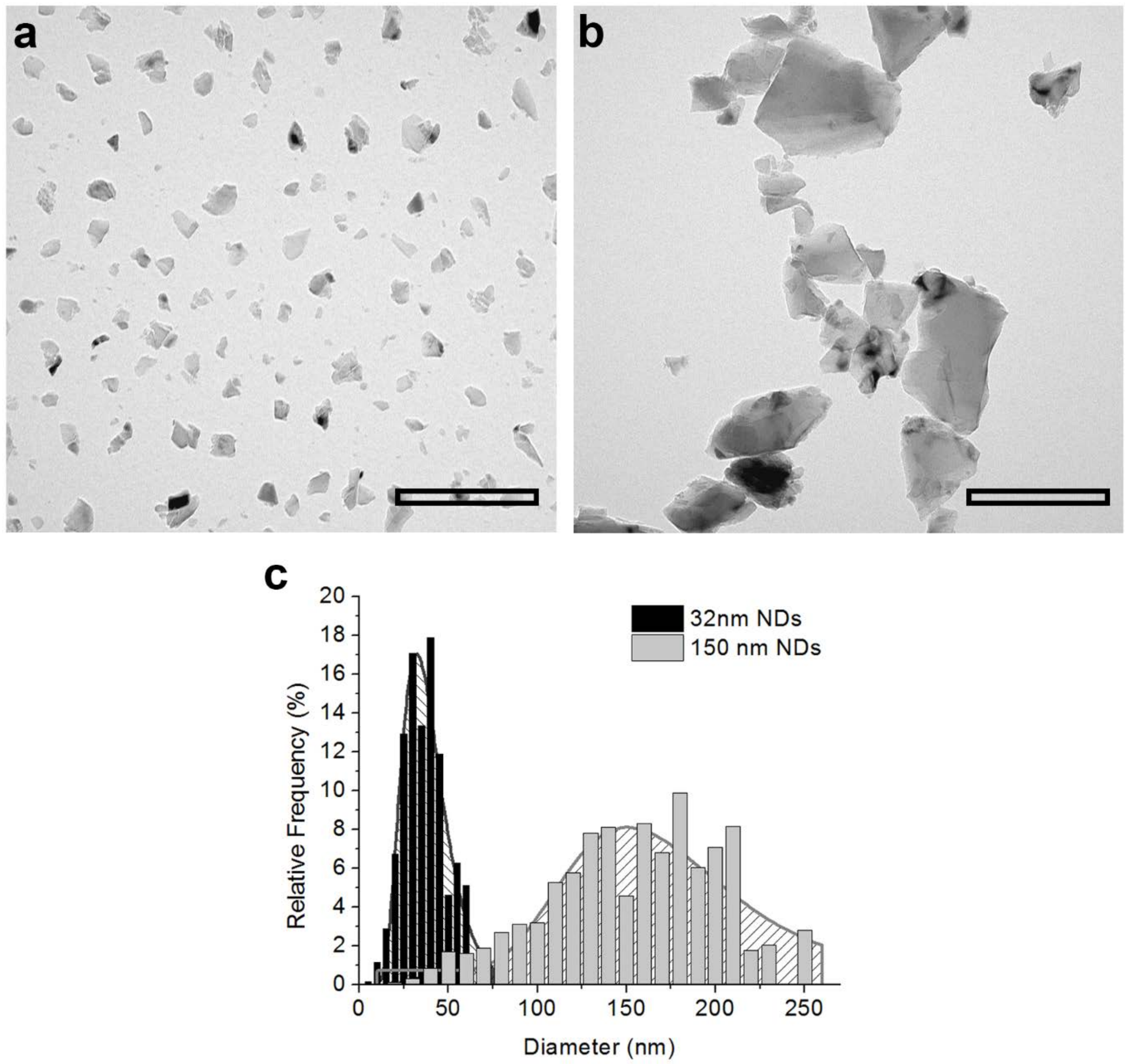

Supplementary Figure 3. Transmission electron microscopy (TEM) of nanodiamonds (NDs). TEM images of (**a**) 35-nm and (**b**) 150-nm fluorescent NDs obtained after isolation and purification from the $^{10}B_2O_3$ melt. The scale bars correspond to 200 nm. (**c**) Size distributions are based on analysis of approximately 1000 particles from each TEM micrograph. Particle size is expressed by equivalent circular diameters recalculated to volume-weighted histograms. Obtained histograms are fitted with a log-normal distribution.

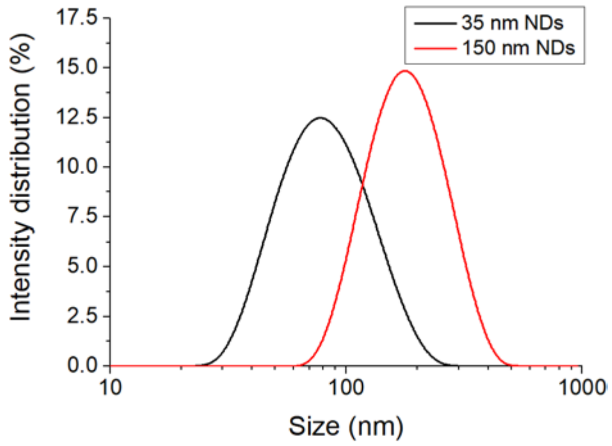

Supplementary Figure 4. Dynamic light scattering (DLS) characterization. Size distribution of 35-nm (black) and 150-nm (red) fluorescent nanodiamonds (NDs). Concentration of the colloids was 0.5 mg ml^{-1}. Measured zeta potentials were –46.7 mV for 35-nm particles and –41.0 mV for 150-nm particles.

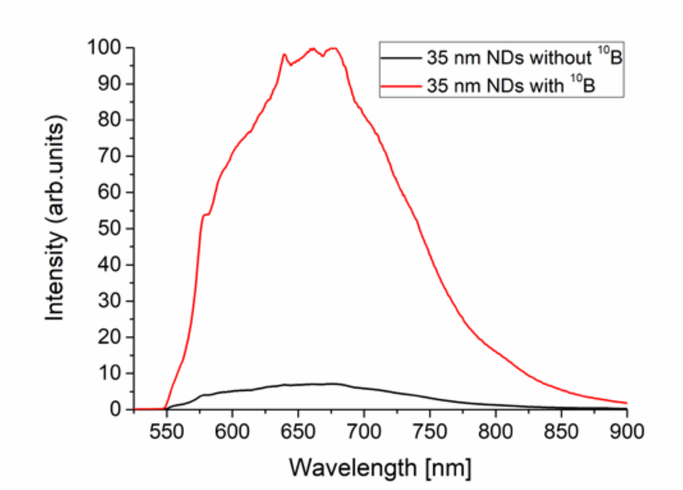

Supplementary Figure 5. Control irradiation without presence of $^{10}B_2O_3$. Comparison of fluorescence intensity of 35-nm nanodiamonds (NDs) irradiated by neutrons only (control sample without presence of $^{10}B_2O_3$) and by neutrons in a melt of $^{10}B_2O_3$. The fluorescence intensity of the sample irradiated with $^{10}B_2O_3$ is 14× higher than that of the sample irradiated without $^{10}B_2O_3$.

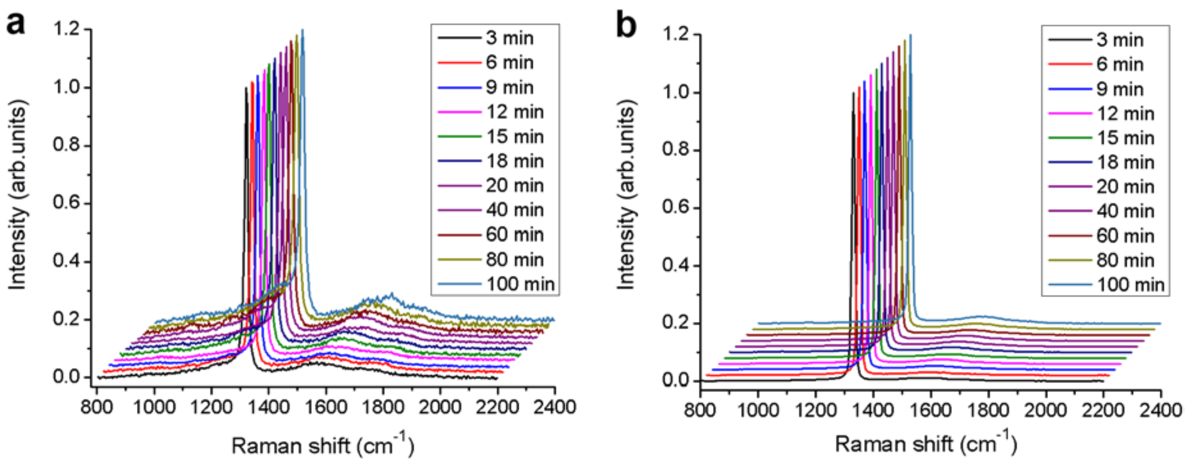

Supplementary Figure 6. Raman spectra of nanodiamonds (NDs). (**a**) 35-nm and (**b**) 150-nm NDs were irradiated with neutrons in a nuclear reactor in melt with $^{10}B_2O_3$ for the indicated durations.

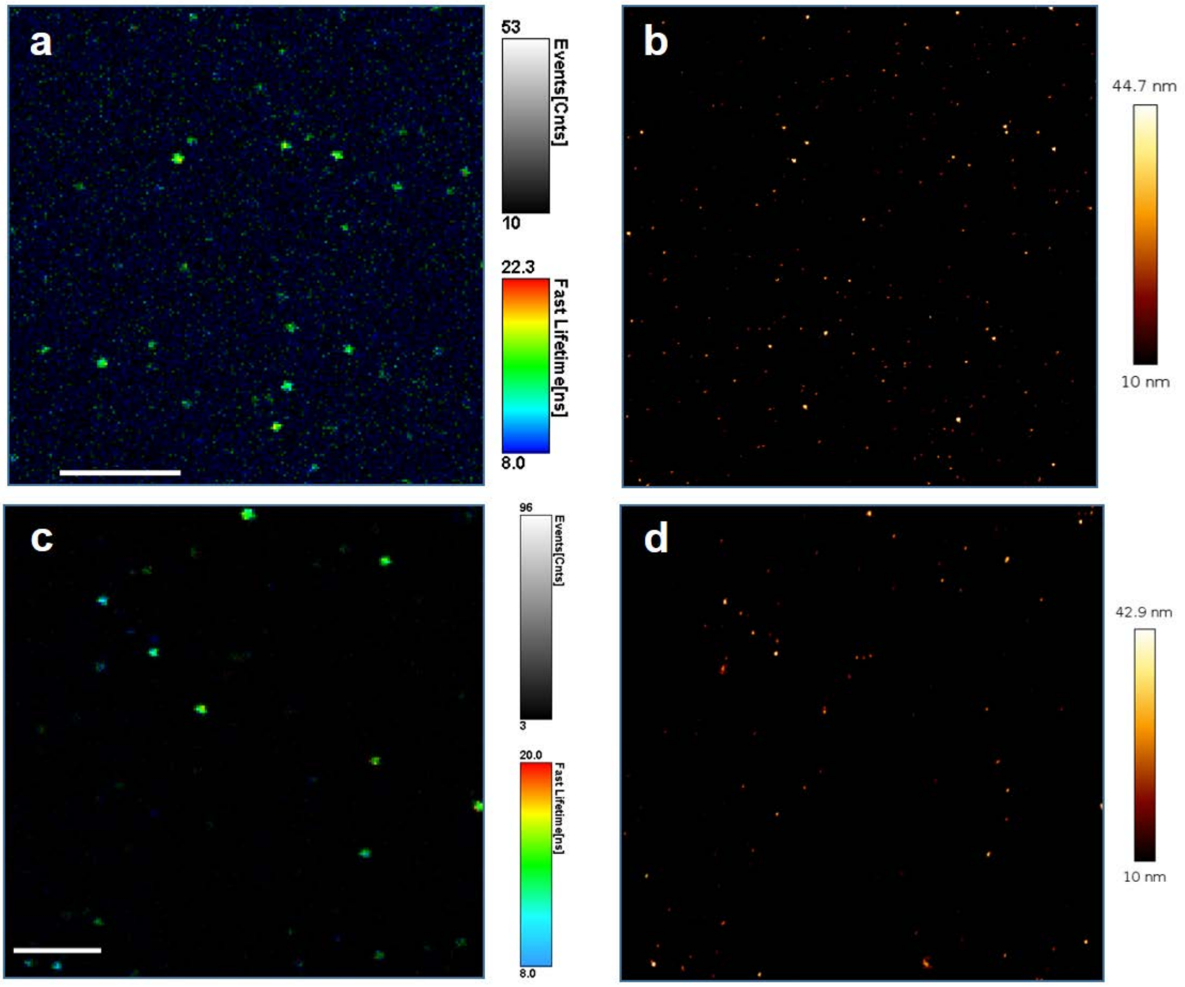

Supplementary Figure 7. Spectral characterization of fluorescent nanodiamonds (FNDs). Representative examples of (**a**, **c**) fluorescence-lifetime imaging (FLIM) and (**b**, **d**) atomic force microscopy (AFM) analysis of FNDs irradiated (**a**, **b**) by p^+ in a cyclotron and (**c**, **d**) by neutrons in a nuclear reactor in melt with $^{10}B_2O_3$. The FNDs were deposited on a glass cover slip, and AFM and FLIM were taken simultaneously. Nanoparticles with diameter below 10 nm and clusters of sized larger than 50 nm were not counted in the overall statistics. The scale bars correspond to (**a**, **b**) 7 µm and (**c**, **d**) 5 µm, respectively.

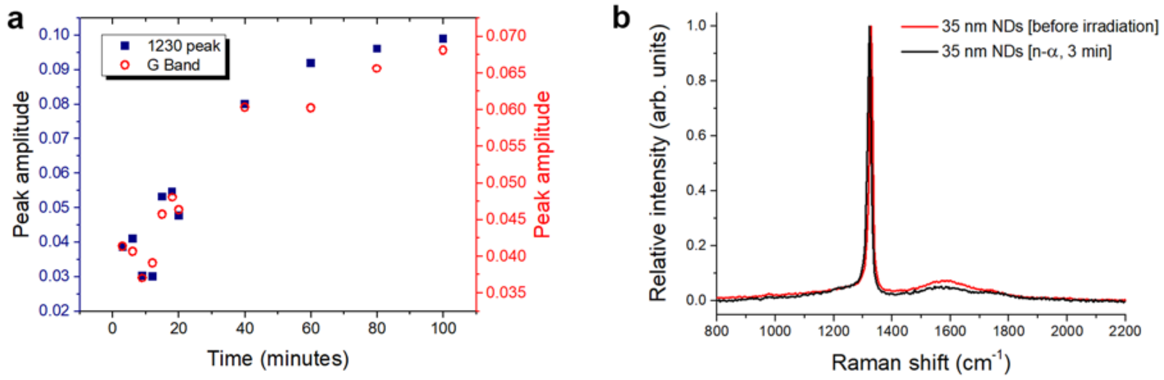

Supplementary Figure 8. Radiation damage of nanodiamonds (NDs). (**a**) Correlation of peak amplitudes in Raman spectra of 35-nm NDs irradiated in a nuclear reactor by neutrons in a $^{10}B_2O_3$ melt. The increase in the peak at ~1230 cm^{-1} corresponds to an increase in the G-band at ~1600 cm^{-1}. (**b**) The Raman spectra of NDs before and after optimized irradiation have comparable intensities at the ~1600 and ~1230 cm^{-1}.

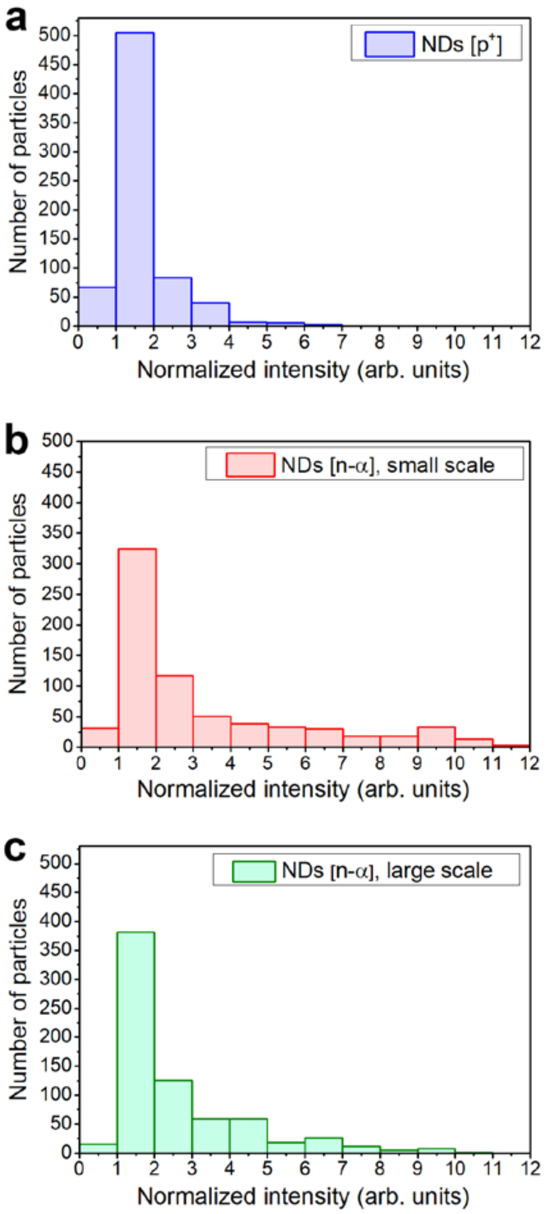

Supplementary Figure 9. Histograms of fluorescence intensity distributions. 35-nm nanodiamonds (NDs) irradiated (**a**) in a cyclotron by protons using optimized pellet target and in a nuclear reactor by neutrons in a $^{10}B_2O_3$ melt at (**b**) a small scale and (**c**) a large scale. Fluorescence intensity was normalized to the average intensity of one NV center in ND particle. The total number of particles was normalized to 1,000.

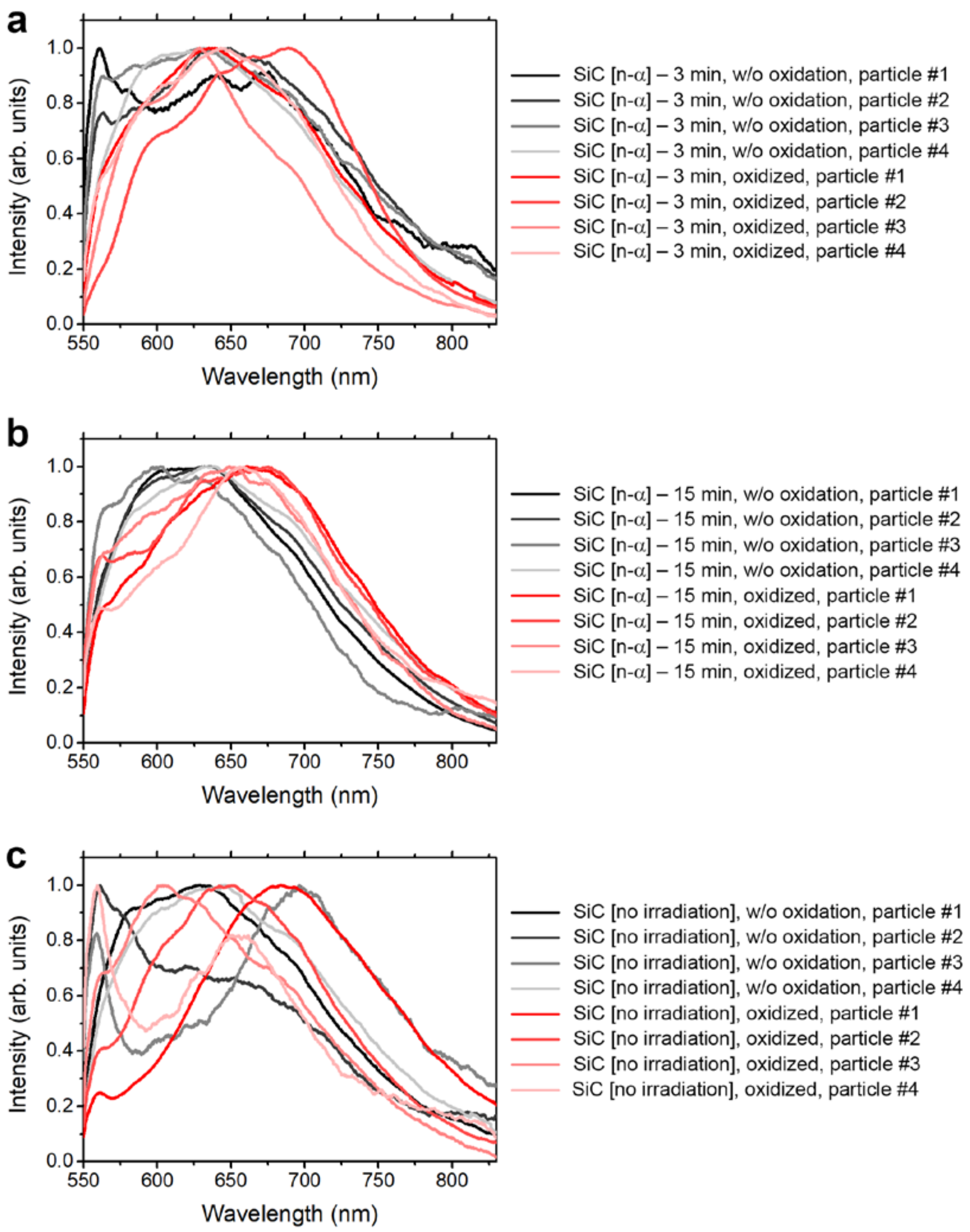

Supplementary Figure 10. Photoluminescence spectra taken from single SiC nanoparticles. Samples (**a**) irradiated for 3 minutes (2.56×10^{-3} dpa), (**b**) irradiated for 15 minutes (1.28×10^{-2} dpa) and (**c**) not irradiated. Each graph compares samples before annealing and oxidation (black to gray) and after oxidation and annealing (red to pink).

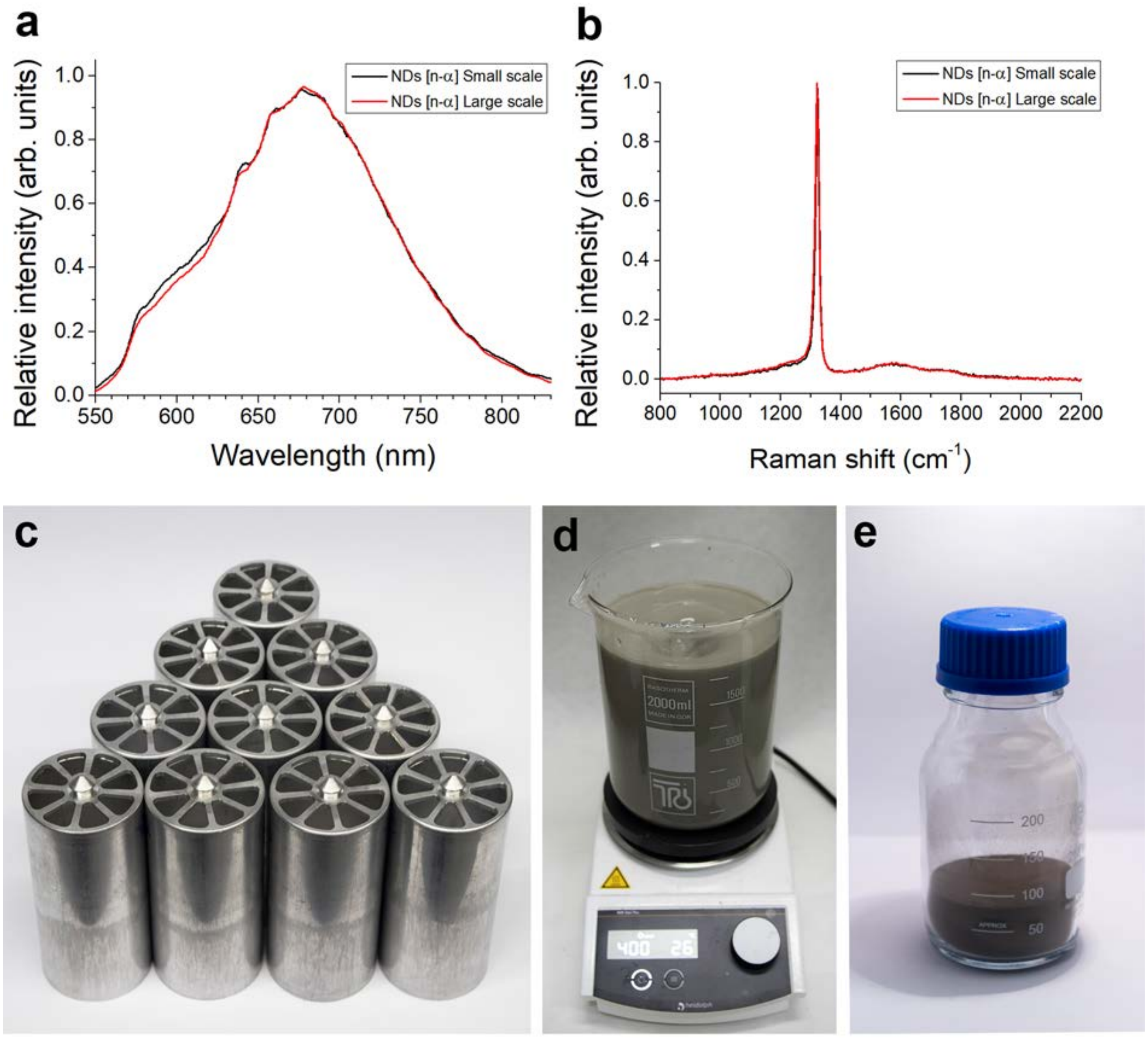

Supplementary Figure 11. Large-scale preparation of fluorescent nanodiamonds (NDs). (**a**, **b**) Spectral characterization of 35-nm NDs irradiated in a nuclear reactor at a small and large scale. (**a**) Photoluminescence spectra normalized to their maxima. (**b**) Raman spectra normalized to the diamond Raman band. The results confirm that the large-scale preparation provided fluorescent NDs with equal quality to the small-scale. (**c**) Ten aluminum capsules designed for large-scale irradiation of the ND-$^{10}B_2O_3$ composite. Each capsule contained 24 g of material sealed in a 1-mm layer between double walls. (**d**) Dispersion of irradiated NDs during aqueous washing. (**e**) Purified fluorescent NDs (70 g) after irradiation, washing and lyophilisation.

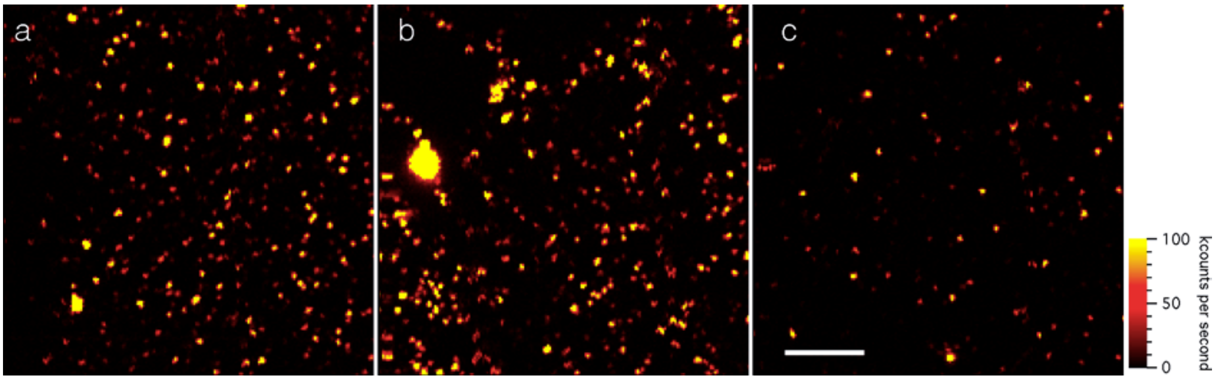

Supplementary Figure 12. Confocal fluorescence images of nanodiamonds (NDs) at 4K. The NDs were dispersed on a silicon wafer substrate in a cryostat. (**a**) Electron-irradiated commercially available NDs (100 nm ND [e$^-$]). (**b**) 150 nm NDs [e$^-$]. (**C**) 150 nm NDs [n-α]. The scale bar corresponds to 20 μm.

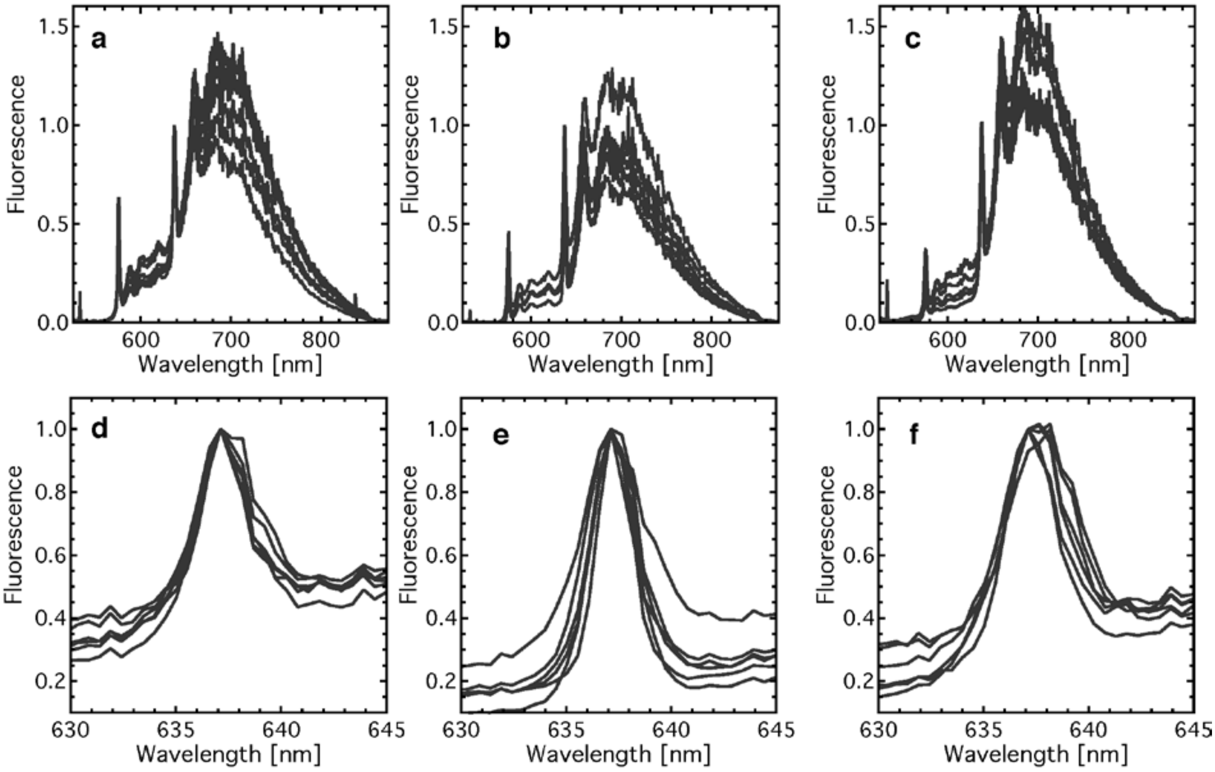

Supplementary Figure 13. Fluorescence spectra of nanodiamonds (NDs) at 4K. The spectra were normalized to the NV$^-$ zero phonon line (ZPL) at 637 nm. (**a**, **d**) Electron-irradiated commercially available NDs (100 nm ND [e$^-$]). (**b**, **e**) 150 nm NDs [e$^-$]. (**c**, **f**) 150 nm NDs [n-α]. (**a**-**c**) Full fluorescence spectra showing the laser excitation (532 nm) and sharp NV^0 and NV$^-$ ZPL emission peaks at 575 nm and 637 nm, respectively. Well-defined phonon side-bands are also visible in all spectra. (**d**-**f**) Zoom into the spectral region of the NV$^-$ ZPLs.

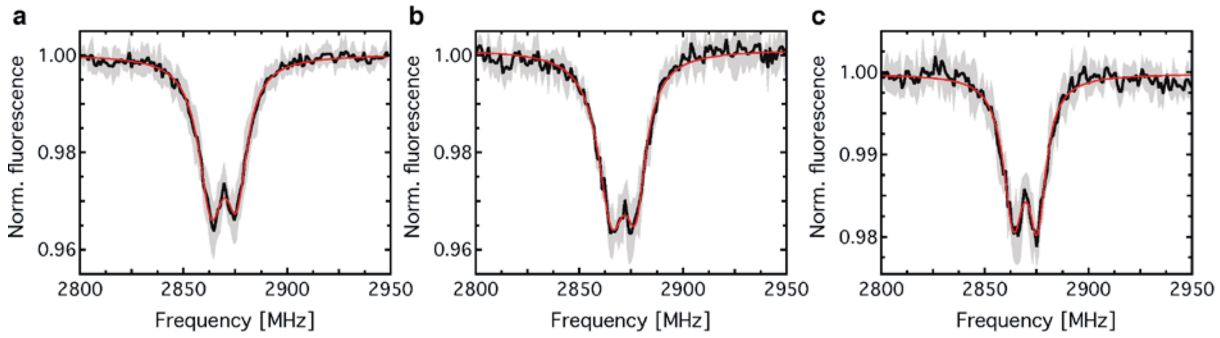

Supplementary Figure 14. Optically detected magnetic resonance spectra of nanodiamonds (NDs). The spectra at zero magnetic field at room temperature of (**a**) electron-irradiated commercially available NDs (100 nm ND [e$^-$]), (**b**) 150 nm NDs [e$^-$], and (**c**) 150 nm NDs [n-α]. All spectra (black solid line) are averages over 5 individual measurements and the standard deviation between individual measurements are indicated by grey areas. The zero-field splitting values given in Figure 3D were determined by double Lorentzian fits to the experimental traces (red lines).
